# A Novel N-Doped Nanoporous Bio-Graphene Synthesized from *Pistacia lentiscus* Gum and Its Nanocomposite with WO_3_ Nanoparticles: Visible-Light-Driven Photocatalytic Activity

**DOI:** 10.3390/molecules26216569

**Published:** 2021-10-29

**Authors:** Maryam Afsharpour, Mehdi Elyasi, Hamedreza Javadian

**Affiliations:** Chemistry & Chemical Engineering Research Center of Iran (CCERCI), Tehran P.O. Box 14335-186, Iran; melyasi@ccerci.ac.ir (M.E.); hamedreza.javadian@yahoo.com (H.J.)

**Keywords:** bio-graphene, nitrogen-doped, tungsten oxide, nanocomposite, photocatalyst, adsorption, methyl red, Congo red, methyl orange, tetracycline, acetaminophen

## Abstract

This paper reports the synthesis of a new nitrogen-doped porous bio-graphene (NPBG) with a specific biomorphic structure, using *Pistacia lentiscus* as a natural carbon source containing nitrogen that also acts as a bio-template. The obtained NPBG demonstrated the unique feature of doped nitrogen with a 3D nanoporous structure. Next, a WO_3_/N-doped porous bio-graphene nanocomposite (WO_3_/NPBG-NC) was synthesized, and the products were characterized using XPS, SEM, TEM, FT-IR, EDX, XRD, and Raman analyses. The presence of nitrogen doped in the structure of the bio-graphene (BG) was confirmed to be pyridinic-N and pyrrolic-N with N1 peaks at 398.3 eV and 400.5 eV, respectively. The photocatalytic degradation of the anionic azo dyes and drugs was investigated, and the results indicated that the obtained NPBG with a high surface area (151.98 m^2^/g), unique electronic properties, and modified surface improved the adsorption and photocatalytic properties in combination with WO_3_ nanoparticles (WO_3_-NPs) as an effective visible-light-driven photocatalyst. The synthesized WO_3_/NPBG-NC with a surface area of 226.92 m^2^/g displayed lower bandgap and higher electron transfer compared with blank WO_3_-NPs, leading to an increase in the photocatalytic performance through the enhancement of the separation of charge and a reduction in the recombination rate. At the optimum conditions of 0.015 g of the nanocomposite, a contact time of 15 min, and 100 mg/L of dyes, the removal percentages were 100%, 99.8%, and 98% for methyl red (MR), Congo red (CR), and methyl orange (MO), respectively. In the case of the drugs, 99% and 87% of tetracycline and acetaminophen, respectively, at a concentration of 10 mg/L, were removed after 20 min.

## 1. Introduction

The environmental impact of industrial wastewaters is a serious concern all around the world [1,2,3,4]. The contaminants circulating in wastewaters are frequently complicated and non-biodegradable, which requires treatment using special removal methods. Different technologies, such as chemical coagulation, adsorption, filtration, reverse osmosis, and catalytic or photocatalytic treatment have been used to remove chemical contaminants [5,6,7,8,9,10,11,12]. In recent years, advanced oxidation processes (AOPs) utilizing catalysts have been applied as efficient methods to eliminate organic pollutants by degrading them into harmless substances via electron-hole generation under light irradiation or in the presence of oxidants [10,11,12,13,14,15,16,17,18,19].

Metal oxides, such as titanium dioxide, zinc oxide, and tungsten oxide, as well as some metal-free compounds, such as g-C_3_N_4_ and SiC, are semiconductors with interesting advantages in removing pollutants via AOPs [20,21,22,23,24,25,26]. Among the photocatalysts used in AOPs, the attractive properties of tungsten oxide have been demonstrated by researchers [27,28,29].

Doping and combining metal oxides with other semiconductors can improve photocatalytic activity by increasing the separation of charge and lowering the recombination rate. Graphene is of significant interest in photocatalytic applications because its excellent electronic properties, high conductivity, and high surface area can increase the transfer of electrons and strengthen photocatalytic capabilities [30,31,32].

In this study, a novel N-doped nanoporous bio-graphene (NPBG) synthesized using *Pistacia lentiscus* as a natural source was used as a substrate to synthesize tungsten oxide nanocomposite as a visible-light-driven semiconductor. Because of its delocalized electrons, which cause the charge transfer, NPBG was chosen to prevent the recombination of produced electrons in the WO_3_ photocatalyst. To characterize the obtained products, XPS, SEM, TEM, FT-IR, EDX, XRD, and Raman were applied. The synthesized nanocomposite was applied for the photocatalytic degradation of the anionic azo dyes and drugs, and the optimum conditions for maximum removal were obtained.

## 2. Materials and Methods

### 2.1. Materials

*Pistacia lentiscus* was used as a natural carbon source containing nitrogen for the synthesis of the NBG. Tungstic acid and hydrogen peroxide (30%) purchased from Merck (Darmstadt, Germany) were used to synthesis the nanocomposite for the photocatalytic degradation process. The azo dyes, including CR (C.I. Direct Red 28), MO (C.I. Acid Orange 52), and MR (C.I. Acid Red 2), and acetaminophen and tetracycline were selected as model drugs to test the photocatalytic activity of the synthesized nanocomposite.

### 2.2. Characterization

The XPS analysis was performed using an ESCALAB 250Xi Thermo Scientific System (Waltham, MA, USA) (MgKα = 1253.6 eV). SEM images were obtained using a TESCAN, VEGA3 microscope (Brno, Czech Republic) equipped with an X-ray energy dispersive spectroscope (EDX). The specific surface area and pore size distribution of the samples were evaluated by means of the BELSORP Mini Instrument (Microtrac MRB, York, PA, USA). Fourier-transform infrared (FT-IR) spectra were recorded by Bruker Vector 33 spectrometer (Leipzig, Germany), and Raman spectra of the samples were obtained using Bruker Senterra micro-Raman (Leipzig, Germany) at 785 nm laser wavelength. The XRD (X-ray diffraction) patterns were recorded with Bruker AXS (Leipzig, Germany) using CuK_α_ radiation with λ = 1.54060 Å. The total organic carbon (TOC) was obtained by a TOC analyzer (Multi N/C 3100, Jena, Germany)

### 2.3. Synthesis of NBG (N-Doped Bio-Graphene) and NPBG (N-Doped Porous Bio-Graphene)

The NBG was synthesized using a natural precursor to obtain the N-doped biomorphic structure. First, *Pistacia lentiscus* gum was ground to obtain a soft powder. Next, the black powder of NBG with a special morphology of graphenic layers was obtained through the carbonization of the soft powder at 700 °C for 1 h in an N_2_ atmosphere with a heating rate of 5 °C/min. As surface area is one of the main parameters in catalytic performance, the porosity of the synthesized NBG was increased through KOH activation. For this purpose, 20 g of KOH was dissolved in 100 mL of distilled water. Next, 10 g of synthesized NBG was added to this solution, and the mixture was stirred at 50 °C for 4 h. The obtained product was dried at 80 °C and then treated at 700 °C for 1 h in an N_2_ atmosphere (5 °C/min).

### 2.4. Synthesis of WO_3_/NPBG-NC (WO_3_/N-Dope Porous Bio-Graphene Nanocomposite)

For the synthesis of WO_3_/NPBG-NC, oxodiperoxo tungsten complex (WO(O_2_)_2_) was used as the tungsten precursor. To prepare the tungsten complex, 10 mmol (2.5 g) of tungstic acid was dissolved in 20 mL of H_2_O_2_ (30%) at 40 °C for 48 h. Next, 1 g of synthesized NPBG was added to the solution and stirred at 80 °C for 24 h to stabilize the WO_3_-NP_S_ on the surface of NPBG. The obtained nanocomposite was filtered, washed several times with distilled water, and finally calcined at 600 °C for 1 h. Since increasing the amount of active site (WO_3_-NPs) in the composite increases the photocatalytic activity, the maximum amount of WO_3_-NPs was stabilized on the surface of NPBG using this method.

To compare the bandgap of WO_3_/NPBG-NC with WO_3_-NPs, WO_3_-NP_S_ were synthesized via the same precursor. The peroxo-tungsten solution (WO(O_2_)_2_) was treated at 80 °C under ultrasonic conditions to prevent the agglomeration of the particles. The obtained powder was calcined at 600 °C for 1 h.

### 2.5. Photocatalytic Degradation Method

The photocatalytic activity of the synthesized nanocomposite was evaluated through the degradation of three dyes (CR, MO, and MR) and two drugs (acetaminophen and tetracycline) under visible-light irradiation. The optimal conditions of the photocatalytic system were obtained by investigating the effect of the initial concentration of dyes (100, 200, and 300 mg/L) and the amount of the nanocomposite (0.01, 0.015, and 0.02 g) at room temperature (25 °C). Before irradiation, the adsorption percentage of the analytes was measured in dark conditions. Next, a certain amount of synthesized photocatalyst was dispersed in 10 mL of pollutants solution under stirring (120 rpm) at visible light irradiation. The photocatalytic experiments were carried out in a glass reactor under air bubbling to saturate the solution with oxygen during the reaction. A Xenon lamp (Autotech H7, Guangzhou, China) was used as a visible light source with a light flux of 30 W/cm^2^. The samples were collected every 10 min from the reaction mixture to determine the concentrations of the pollutants. After centrifugation and filtration of the photocatalyst, the samples were analyzed using a UV-Vis spectrophotometer (Perkin-Elmer Lambda 35) (Seer Green, UK) at 497 nm, 464 nm, 425 nm, 246 nm, and 357 nm to measure the concentrations of CR, MO, MR, acetaminophen, and tetracycline, respectively.

The removal percentage of the pollutant was measured using the following equation:

(1) $$\mathrm{Removal}\;\%=\left[\frac{\left(C_{0}-C_{t}\right)}{C_{0}}\right]*100\%$$

where *C*_0_ and *C_t_* are the initial and equilibrium concentrations of the pollutants, respectively.

To investigate the degradation mechanism, quenching tests were performed using 2 mM of isopropanol (IPA), 1,4-benzoquinone (BQ), ammonium oxalate (AO) as the hydroxyl radical (^•^OH), superoxide radical anions (^•^O_2_^−^), and hole (h^+^) scavenger, respectively.

The reusability of a photocatalyst is considered an important parameter in practical applications. Therefore, it was studied by performing five cycles after centrifuging and washing the photocatalyst with distilled water and drying at 80 °C.

## 3. Results and Discussion

### 3.1. Characterization

**Figure 1:**
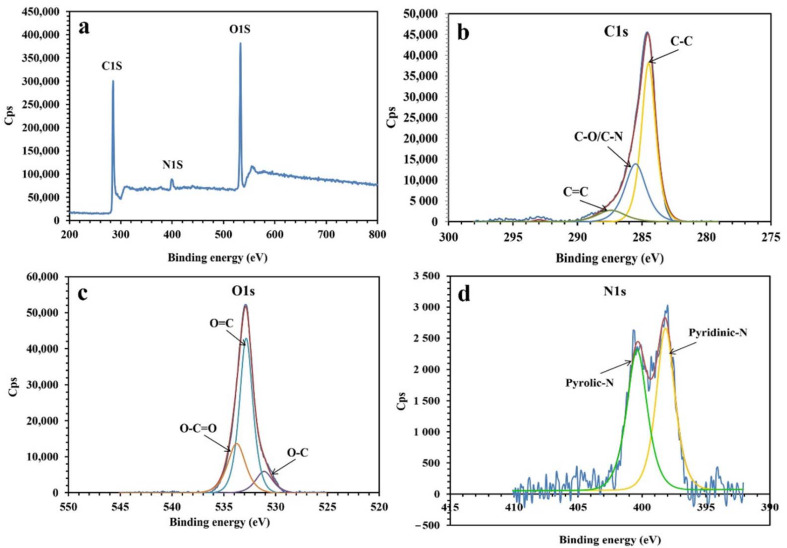
XPS analysis of NPBG: (**a**) XPS survey, (**b**) C1s, (**c**) O1s, and (**d**) N1s.

Figure 1 presents the XPS analysis of the synthesized NPBG. The XPS spectra present the photoemission peaks of C1s, O1s, and N1s, indicating the presence of nitrogen atoms doped in the graphene structure, originating from the natural gum. As shown in Figure 1d, the spectrum of N1s is deconvoluted into two peaks at 398.3 eV and 400.5 eV, indicating pyridinic-N and pyrrolic-N species in the structure of NPBG. The spectrum of C1s presents three peaks at 284.5 eV, 285.5 eV, and 287.4 eV, corresponding to C-C, C-O/C-N, and C=O bonds, respectively. As for the spectrum of O1s, it displays O-C, O=C, and O-C=O bonds at 531.3 eV, 532.9 eV, and 533.8 eV, respectively. The XPS results also demonstrate the amount of elements to be 75.87, 21.42, and 2.71 At% for C, O, and N, respectively.

**Figure 2:**
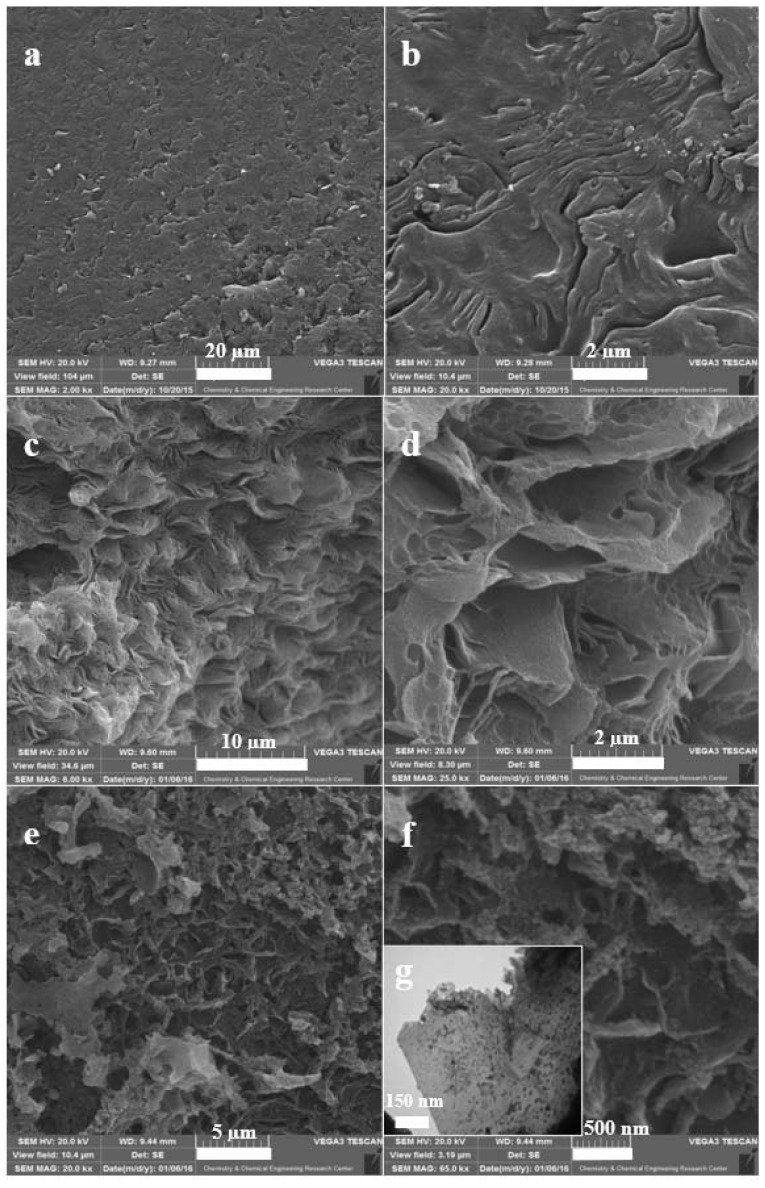
SEM images of (**a**,**b**) NBG, (**c**,**d**) NPBG, (**e**,**f**) WO_3_/NPBG-NC; (**g**) TEM image of WO_3_/NPBG-NC.

Figure 2 presents the SEM images of synthesized NBG, NPBG, and WO_3_/NPBG-NC. Figure 2a,b present the SEM images of synthesized NBG that display the packed layers. Figure 2c,d display the morphology of NPBG that indicates the separated layers of graphene. This special morphology of graphenic layers originates in natural *Pistacia lentiscus* gum. In the SEM images of the WO_3_/NPBG-NC shown in Figure 2e,f as well as its TEM image (Figure 2g), WO_3_-NPs can be clearly observed on the surface of the graphenic layers.

**Figure 3:**
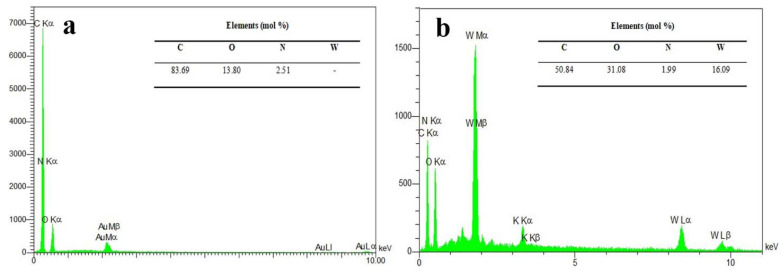
EDX spectra of (**a**) NPBG and (**b**) WO_3_/NPBG-NC.

Figure 3 presents the EDX spectra of the NPBG and WO_3_/NPBG-NC and their elemental amounts, confirming the presence of 2.51% of N doped in the synthesized NPBG and 16.09% of tungsten in the nanocomposite. These percentages are close to those obtained bythe XPS analysis (Figure 1).

**Figure 4:**
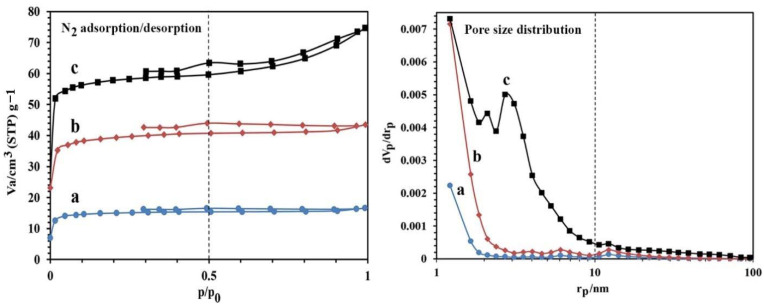
N_2_ adsorption/desorption and pore size distribution of (**a**) NBG, (**b**) NPBG, and (**c**) WO_3_/NPBG-NC.

The values of porosity obtained for the synthesized NBG, NPBG, and WO_3_/NPBG-NC are reported in Figure 4 and Table 1. The results show that the surface area of the NBG increased from 38.98 m^2^/g to 151.98 m^2^/g after the activation process with KOH. This increase can also be observed in the SEM images. As shown in Table 1, the surface area of the WO_3_/NPBG-NC (226.92 m^2^/g) is higher than that of the NPBG due to the immobilization of the WO_3_-NPs on the surface of the NPBG. The values of average pore diameter are 1.77 nm and 6.68 nm, respectively, for the NPBG and WO_3_/NPBG-NC, indicating the microporous structure of the NPBG and the mesoporous structure of the nanocomposite.

**Figure 5:**
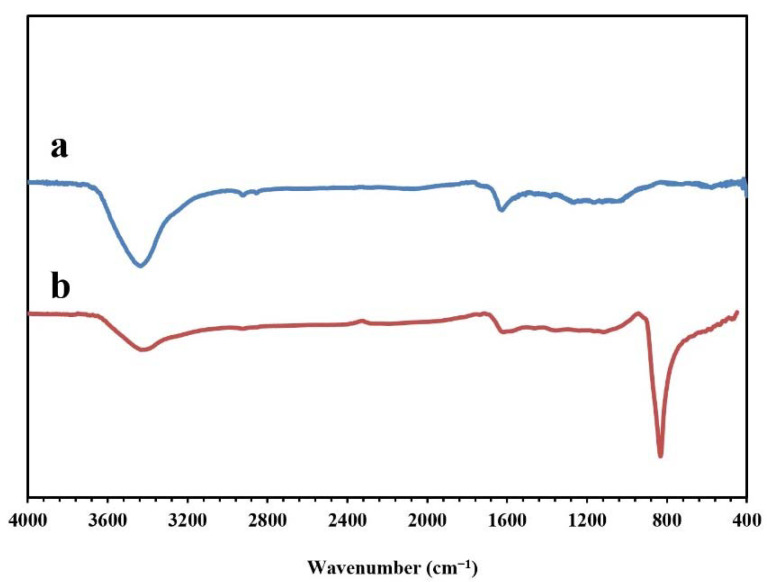
FT-IR spectra of (**a**) NPBG and (**b**) WO_3_/NPBG-NC.

Figure 5 presents the FT-IR spectra of the synthesized NPBG and WO_3_/NPBG-NC. The absorption peaks at around 1096 cm^−1^, 1643 cm^−1^, and 1720 cm^−1^ indicate the stretching vibrations of the C-O, C=C, and C=O groups, respectively. The weak peak at around 1430 cm^−1^ represents the C-N stretching vibration, which confirms nitrogen doping in this structure of the sample. The symmetric and asymmetric vibrations of C-H were observed at around 2850 cm^−1^ and 2928 cm^−1^. In addition, the wide peak at around 3425 cm^−1^ corresponds to the O-H and N-H vibrations. In the spectrum of the WO_3_/NPBG-NC, the new peak that was detected at 842 cm^−1^ was associated with W=O vibration on the tungsten oxide, which confirmed the successful synthesis of the nanocomposite.

**Figure 6:**
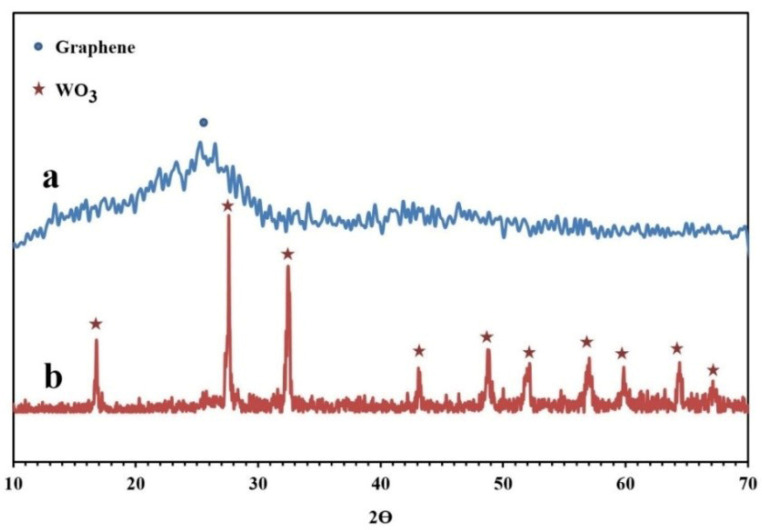
XRD patterns of (**a**) NPBG and (**b**) WO_3_/NPBG-NC.

The XRD patterns of the synthesized NPBG and WO_3_/NPBG-NC are depicted in Figure 6. The XRD pattern of the NPBG displays two diffraction peaks at 2θ = 25.6° and 43.2° that were assigned to the (002) and (100) planes of the carbon phase in the NPBG, respectively. In comparison with the undoped graphene, the major peak is weaker and observed at a higher 2θ (25.6° (doped) vs. 22.3° (undoped)) [33]. The XRD pattern of WO_3_/NPBG-NC displays the appearance of diffraction peaks at 2θ = 16.6°, 27.5°, 32.3°, 43.1°, 48.8°, 51.0°, 56.7°, 59.0°, and 64.2°, which were assigned to (020), (111), (200), (122), (202), (222), (232), (331), and (351) planes of the orthorhombic WO_3_.H_2_O (JCPDS 84-0886).

**Figure 7:**
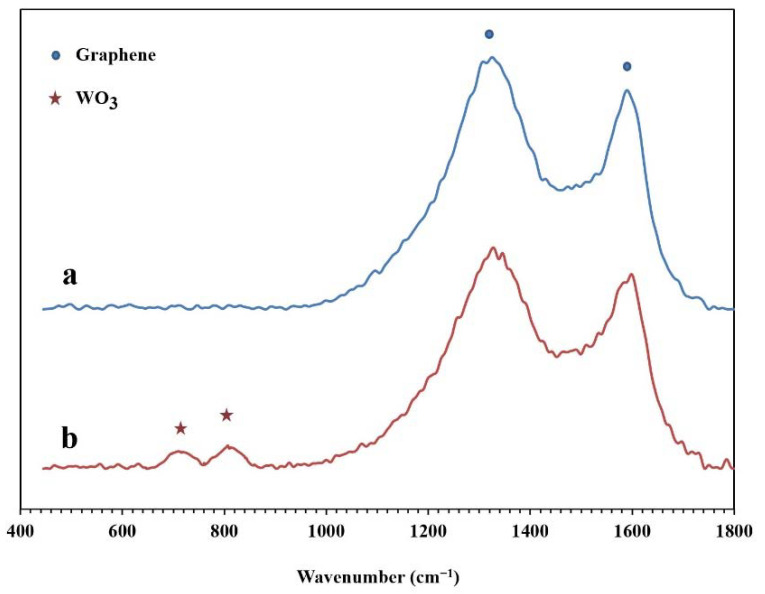
Raman spectra of (**a**) NPBG and (**b**) WO_3_/NPBG-NC.

The Raman spectra of the synthesized NPBG and WO_3_/NPBG-NC are presented in Figure 7. In both samples, two specific peaks of graphene are displayed at around 1330 cm^−1^ and 1580 cm^−1^, which are related to D and G bonds, respectively. In the WO_3_/NPBG-NC, these peaks display a slight shift to higher frequencies, and the new peaks at around 710 cm^−1^ and 805 cm^−1^ confirm the presence of tungsten oxide in the nanocomposite [34].

**Figure 8:**
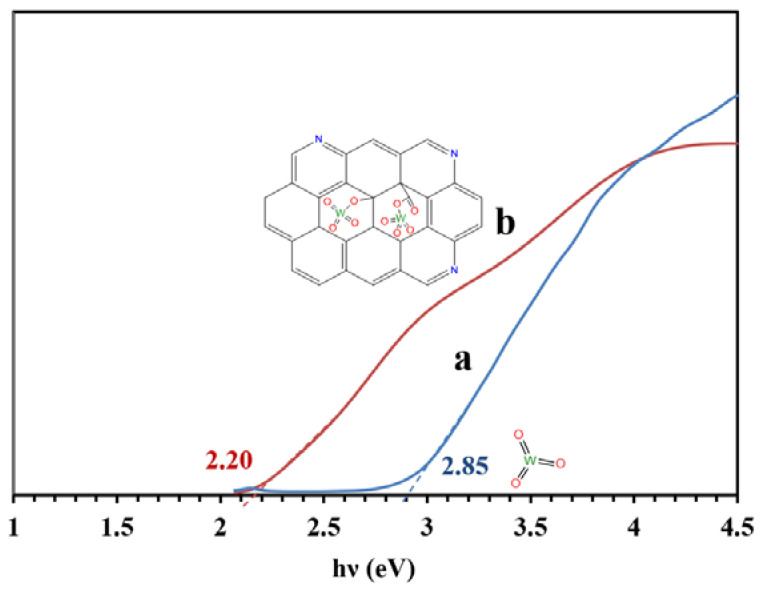
Bandgaps of (**a**) WO_3_-NPs and (**b**) WO_3_/NPBG-NC, according to the Tauc plot.

Figure 8 presents the bandgaps of the WO_3_/NPBG-NC and tungsten oxide calculated from the UV-Vis drift reflectance spectra using the Tauc plot. The results reveal a decrease in bandgap from 2.85 eV to 2.20 eV, owing to the composition of the tungsten oxide with the NPBG.

The valence band (E_VB_) and conduction band (E_CB_) edge potentials can be calculated by the following equations [35]:

E_VB_ = *χ* − E_e_ + 0.5E_g_ (2)

E_CB_ = E_VB_ − E_g_ (3)

where *χ* is the electronegativity and E_g_ is the bandgap energy of the semiconductor. E_e_ is the energy of free electrons vs. the hydrogen scale (4.5 eV). The values of *χ* calculated for the WO_3_-NPs and the WO_3_/NPBG-NC were 6.59 eV and 6.39 eV, respectively [36]. The E_VB_ and E_CB_ values for the WO_3_-NPs were 3.51 eV and 0.66 eV, respectively, while, according to these equations, the E_VB_ and E_CB_ for the WO_3_/NPBG-NC were 2.99 eV and 0.70 eV, respectively. 

### 3.2. Photocatalytic Study

**Figure 9:**
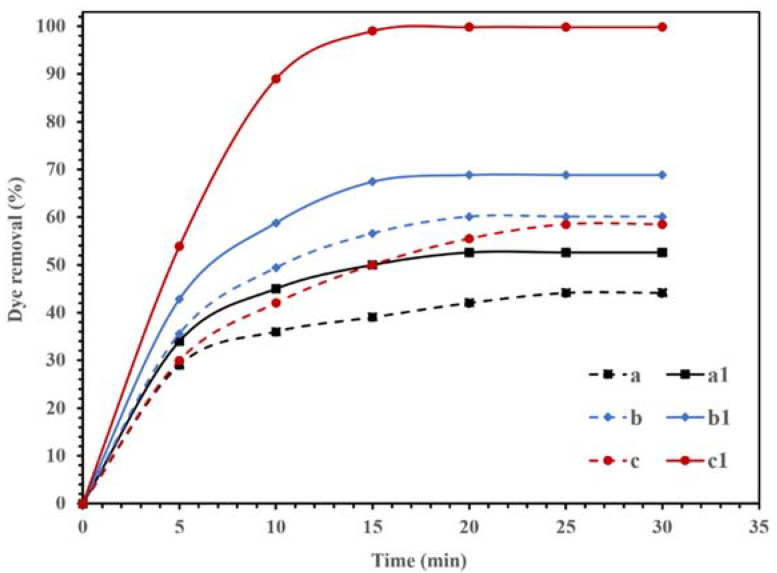
Degradation of 100 mg/L of CR solution using 0.015 g of (**a**,**a1**) NBG, (**b**,**b1**) NPBG, and (**c**,**c1**) WO_3_/NPBG-NC in the dark condition (- - -) and visible light (—).

The photocatalytic activities of the synthesized NBG, NPBG, and WO_3_/NPBG-NC are displayed in Figure 9. The adsorption of CR on the surface of the synthesized materials was recorded in dark conditions, and the results in Figure 9 (dash lines) display the removal of the dye, even in dark conditions, using all the compounds. Graphene is an effective adsorbent due to its high surface area and unique electronic properties [37,38,39,40]. The electrostatic forces and π–π stacking in the graphene structure could be responsible for enhancing the adsorption capacity [40,41,42,43,44]. In addition, the oxygenated functional groups could be effective at potential adsorption [45]. Furthermore, heteroatom-doped graphene shows an improved adsorption performance due to the surface modification [46,47]. As displayed in Figure 9, the synthesized NBG demonstrated 44.1% dye removal in dark conditions, while the NPBG exhibited a higher removal percentage of dye (60.2%) due to its higher surface area. The WO_3_/NPBG-NC demonstrated higher adsorption capacity than the NBG support, which was due to the higher surface area and different surface charges of the nanocomposite.

According to other studies [33,48], the difference in the electronegativity of C and N atoms in the structure of NBG causes a positive charge in C atoms. These positively charged C atoms in the structure of NBG can adsorb O_2_ and produce reactive oxygenated radical species that can decompose the dye, even in dark conditions [48]. 

In WO_3_/NPBG-NC, the immobilization of WO_3_-NPs on the surface of NBG can also change the surface charge, which increases the surface adsorption via the terminal oxygen of WO_3_-NPs. It causes a change in the value of pH_zpc_ from 8.24 for NPBG to 7.1 for WO_3_/NPBG-NC. 

**Figure 10:**
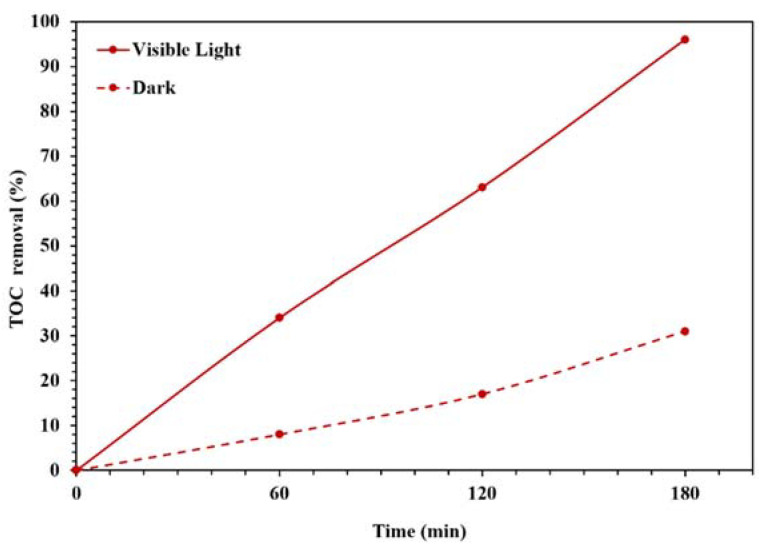
TOC removal efficiency under dark and light irradiation conditions for degradation of 100 mg/L of CR solution using 0.015 g of the photocatalyst.

Figure 9 also displays the photocatalytic performances of synthesized NBG, NPBG, and WO_3_/NPBG-NC. As can be seen, light irradiation can activate the catalyst and enhance the dye removal via the photocatalytic mechanism [20]. The TOC removal of CR solution was also studied under dark and light irradiation conditions (Figure 10). The WO_3_/NPBG-NC demonstrated 99.8% decoloration and 96% reduction in carbon content under light irradiation. The TOC removal of the dye solution was near to the decoloration, indicating the complete degradation of the dye under light irradiation. The results of the decoloration and TOC removal in the dark revealed 58.5% and 31% dye removal, respectively. These results confirm a the presence of a degradation mechanism in addition to the adsorption described above. 

To justify the observed results after the light irradiation, first, the photocatalytic mechanism of WO_3_/NPBG-NC was evaluated.

**Figure 11:**
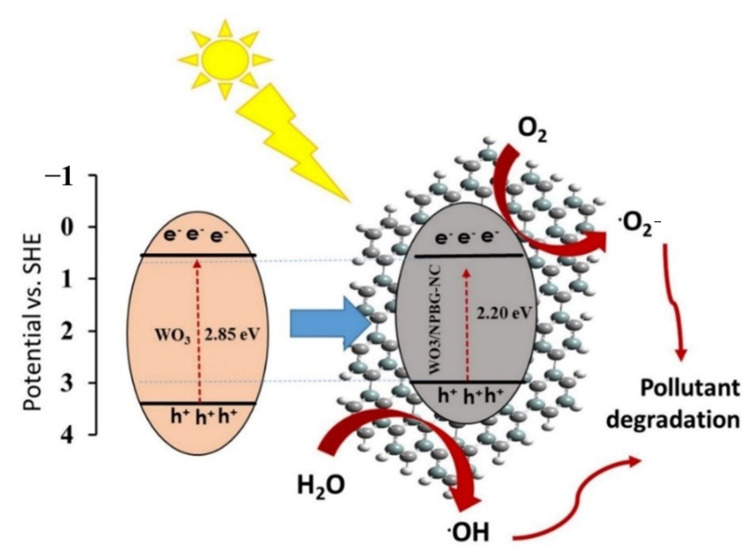
Schematic of photocatalytic mechanism of pollutant degradation by WO_3_/NPBG-NC.

Figure 11 represents the schematic of the proposed photocatalytic mechanisms of the WO_3_-NPs and the WO_3_/NPBG-NC. Under visible light irradiation, the electron-hole pairs in the WO_3_-NPs were generated by the transfer of the generated electrons from the valance bond (VB) to the conduction bond (CB) of the photocatalyst. Next, the excited electrons on the CB were scavenged by NPBG, which that reduced the recombination rate of the electron-hole pairs. The electrons reacted with oxygen to form superoxide radicals, and ^•^OH radical formation took place through reacting holes with H_2_O. The generation of the oxygenated radicals decomposed the organic pollutants into harmless products.

Tungsten oxide is a visible-light-driven semiconductor that exhibits low photocatalytic activity due to its poor charge separation efficacy. Thus, the composition of WO_3_-NPs with NPBG can enhance its photocatalytic properties due to its delocalized electrons, which lead to the charge transfer and prevent the recombination of the generated electrons in the photocatalyst [49,50,51]. NPBG can reduce the bandgap by forming a new valence band and enhancing the charge separation efficiency. It acts as an electron scavenger to accept electrons from WO_3_-NPs, which consequently decreases the electron–hole pair recombination rate. Thus, higher photocatalytic performance is observed with WO_3_/NPBG-NC (Figure 9).

**Figure 12:**
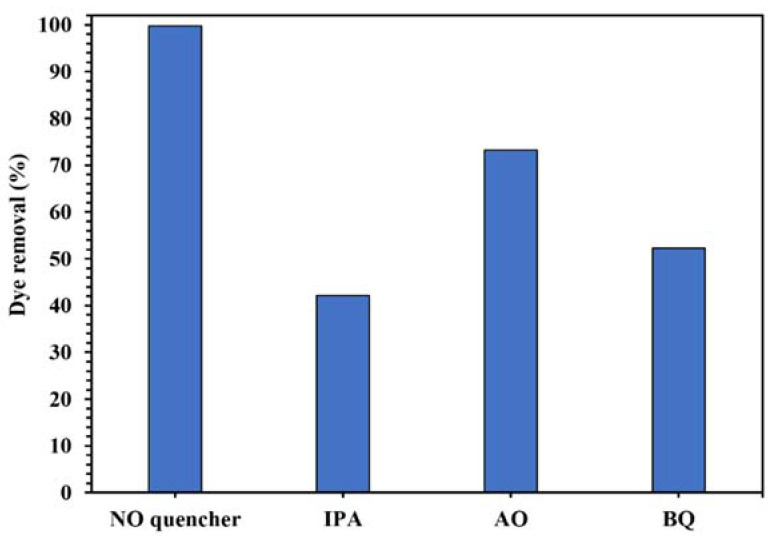
Control experiments of the photocatalytic removal of 100 mg/L of CR solution with the addition of different radical quenchers using 0.015 g of WO_3_/NPBG-NC.

The active oxygenated radical species involved in this photocatalytic process was identified by the quenching experiments. The results in Figure 12 show the decrease of photodegradation of CR from 99.8% to 42.1%, 73.3%, and 52.3% in the presence of IPA, AO, and BQ as ^•^OH, h^+^, and ^•^O_2_^−^ scavenger, respectively, indicating that ^•^OH was the main active radical in the photocatalytic degradation of the dye using WO_3_/NPBG-NC.

**Figure 13:**
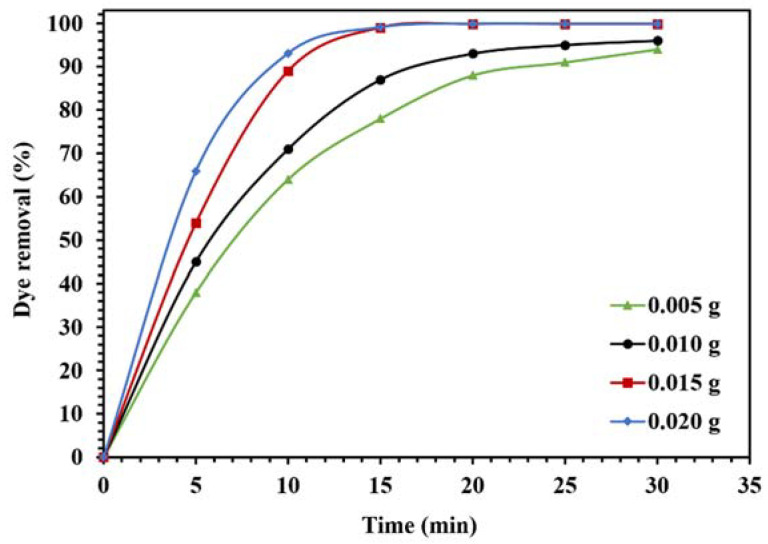
Effect of different amounts of WO_3_/NPBG-NC on the removal of 100 mg/L of CR solution under visible light.

**Figure 14:**
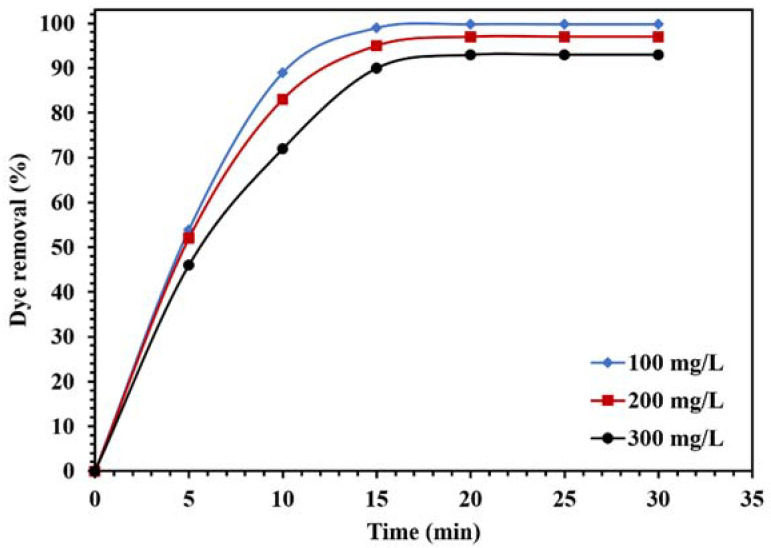
Effect of different concentrations of CR on the photocatalytic activity of 0.015 g of WO_3_/NPBG-NC under visible light.

The optimization of photocatalytic reaction conditions for the degradation of CR in visible light is shown in Figure 13 and Figure 14. To investigate the effect of the amount of photocatalyst on the catalytic activity of WO_3_/NPBG-NC, the degradation of CR was evaluated using different amounts of the nanocomposite (Figure 13). The results demonstrate an increase in dye removal due to increase in the amount of the nanocomposite. Actually, an increase in the photocatalytic active sites occurs, consequently, the oxygenated radicals increase and the degradation process is performed effectively. The optimum amount of the nanocomposite for the maximum removal of the dye was 0.015 g, and a further increase in the amount of the nanocomposite had an effect on the reaction rate (Figure 13).

Figure 14 demonstrates the effect of the dye concentration on the photocatalytic performance of the WO_3_/NPBG-NC. The results in Figure 14 demonstrate that the degradation of the dye decreased along with any increase in the concentration of the dye. The reduction in dye removal at the concentration of 300 ppm can be attributed to the saturation of the catalyst surface with the dye molecules, which caused a decrease in photocatalytic performance.

**Figure 15:**
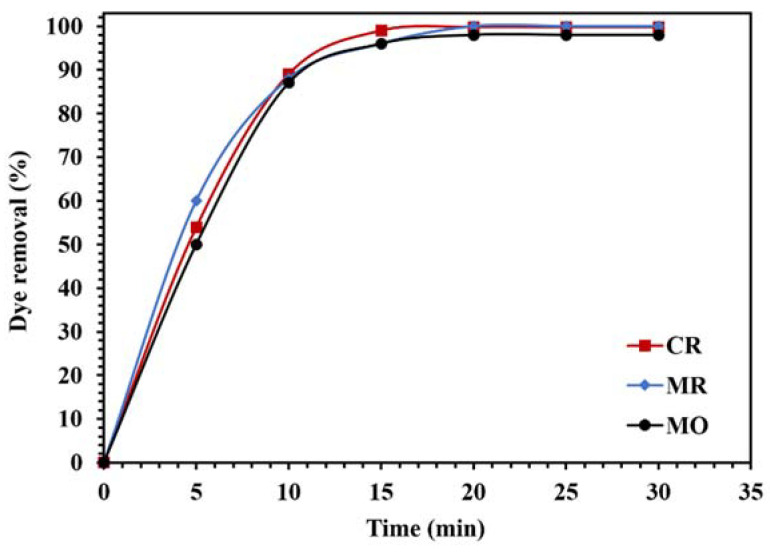
Comparison of the photocatalytic activity of 0.015 g of WO_3_/NPBG-NC for the removal of 100 mg/L of different anionic azo dyes under visible light.

**Figure 16:**
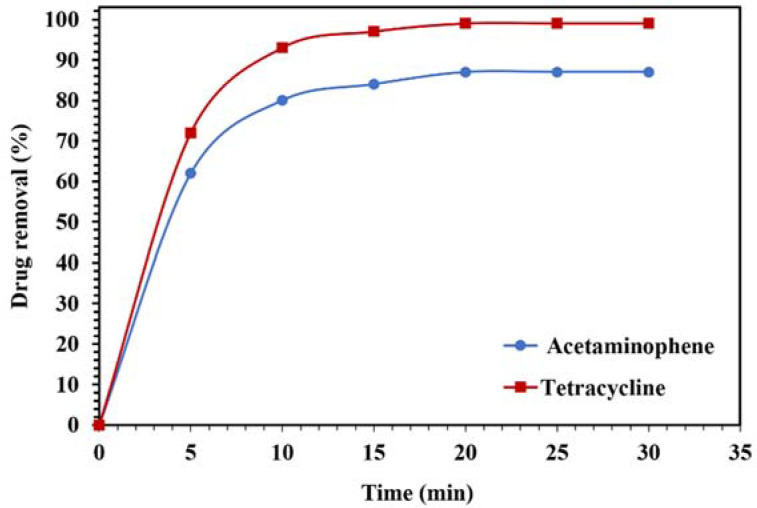
Degradation of 10 mg/L of acetaminophen and tetracycline solutions using 0.015 g of WO_3_/NPBG-NC under visible light.

To investigate the photocatalytic performance of the WO_3_/NPBG-NC for the degradation of different organic pollutants, the removal of different dyes and drugs was tested (Figure 15 and Figure 16). As demonstrated in Figure 15, the WO_3_/NPBG-NC removed 100%, 99.8%, and 98% of the MR, CR, and MO after 15 min, respectively, which indicates the favorable photocatalytic properties of the synthesized nanocomposite for the degradation of the tested azo dyes.

The photodegradation results of the acetaminophen and tetracycline under visible light are displayed in Figure 16. Around 99% and 87% of the tetracycline and acetaminophen, respectively, were removed after 20 min, confirming the suitability of the nanocomposite for the removal of drugs from aqueous media.

**Figure 17:**
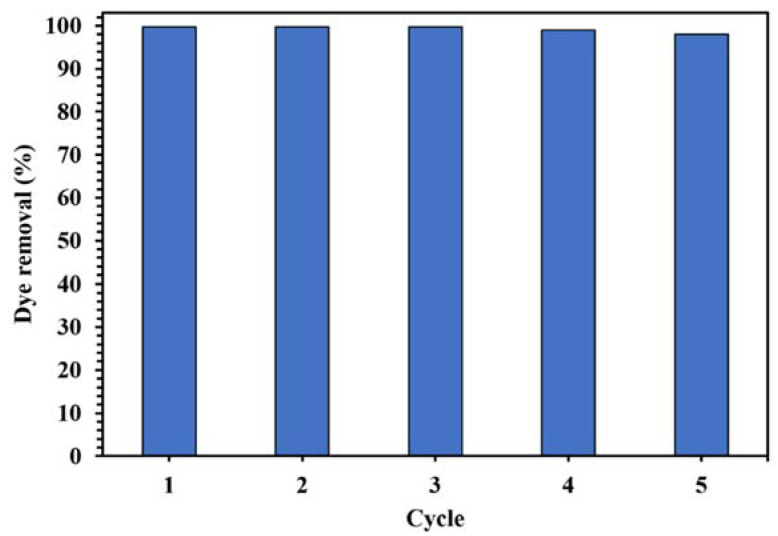
Photocatalytic activity of 0.015 g of WO_3_/NPBG-NC in degradation of 100 mg/L of CR solution after 15 min under visible light for five cycles.

The stability and reusability of a catalyst is an important parameter in designing a suitable catalytic degradation process. Figure 17 displays the cycles of using WO_3_/NPBG-NC for the degradation of CR. As shown in Figure 17, only a slight decrease in the photodegradation of the dye was observed after five cycles, indicating the good reusability of the synthesized nanocomposite in the photocatalytic process.

## 4. Conclusions

In this study, WO_3_/NPBG-NC with an excellent photocatalytic activity was synthesized using WO_3_-NPs and a novel NPBG synthesized from *Pistacia lentiscus* as a natural source. Tungsten oxide is a visible-light-driven semiconductor that exhibits low photocatalytic performance due to its poor charge separation efficacy. The composition of WO_3_-NPs with synthesized NPBG enhanced the photocatalytic activity. The bandgap of the WO_3_-NPs was 2.85 eV, while its value for the WO_3_/NPBG-NC was 2.20 eV. As compared with blank WO_3_, a significant increase in photocatalytic activity was observed using WO_3_/NPBG-NC for the degradation of MR, CR, MO, tetracycline, and acetaminophen, with removal percentage values of 100%, 99.8%, 98%, 99%, and 87%, respectively. The enhancement was due to the fact that the combination of WO_3_ with NPBG improved the photocatalytic property by decreasing the electron–hole recombination rate. The results of the reusability studies revealed that after using WO_3_/NPBG-NC in five cycles, the change in the removal percentage of the dye was negligible, confirming that it can be used as a reusable nanocomposite for the successful degradation of dyes and drugs from aqueous media.

## Figures and Tables

**Table 1:** Textural characterization of the synthesized samples.

Sample	S_BET_ (m^2^/g)	Total Pore Volume (cm^3^/g)	Average Pore Diameter (nm)
**NBG**	38.98	0.027	2.65
**NPBG**	151.98	0.067	1.77
**WO_3_/NPBG-NC**	226.92	0.095	6.68

## Data Availability

Data are contained within the article.

## Sample Availability

Samples of the compounds **1**–**3** are available from the authors.
